# The Protective Effect of Dexmedetomidine Against Ischemia-Reperfusion Injury after Hepatectomy: A Meta-Analysis of Randomized Controlled Trials

**DOI:** 10.3389/fphar.2021.747911

**Published:** 2021-10-12

**Authors:** Ya-Qun Huang, Rui-Ting Wen, Xiao-Tong Li, Jiao Zhang, Zhi-Ying Yu, Yu-Fei Feng

**Affiliations:** ^1^ Department of Pharmacy, Peking University People’s Hospital, Beijing, China; ^2^ Department of Pharmaceutical Administration and Clinical Pharmacy, School of Pharmaceutical Sciences, Peking University Health Science Center, Beijing, China; ^3^ Department of Pharmacy, Peking University Third Hospital, Beijing, China

**Keywords:** hepatectomy, ischemia-reperfusion injury, dexmedetomidine, meta-analysis, randomized controlled trials

## Abstract

**Background:** Hepatic inflow occlusion proceeded to reduce blood loss during hepatectomy induces ischemia-reperfusion (IR) injury in the remnant liver. Dexmedetomidine, a selective α_2_-adrenoceptor agonist used as an anesthetic adjuvant, has been shown to attenuate IR injury in preclinical and clinical studies. However, a meta-analysis is needed to systematically evaluate the protective effect of perioperative dexmedetomidine use on IR injury induced by hepatectomy.

**Methods:** A prospectively registered meta-analysis following Cochrane and PRISMA guidelines concerning perioperative dexmedetomidine use on IR injury after hepatectomy was performed *via* searching Cochrane Library, PubMed, EMBASE, ClinicalTrials.gov, Web of Science, CNKI, WanFang, and Sinomed for eligible randomized controlled trials up to 2021.3.31. The main outcome is postoperative liver function. Risk of bias was assessed by the Cochrane Risk of Bias tool. Review Manager 5.3 and Stata12.0 were applied to perform data analyses.

**Results:** Eight RCTs enrolling 468 participants were included. Compared with 0.9% sodium chloride, dexmedetomidine decreased serum concentration of ALT (WMD = −66.54, 95% CI: −92.10–−40.98), AST (WMD= −82.96, 95% CI: −106.74–−59.17), TBIL (WMD = −4.51, 95% CI: −7.32–−1.71), MDA (WMD = −3.09, 95% CI: −5.17–−1.01), TNF-α (WMD = −36.54, 95% CI: −61.33–−11.95) and IL-6 (WMD = −165.05, 95% CI: −225.76–−104.34), increased SOD activity (WMD = 24.70, 95% CI: 18.09–31.30) within postoperative one day. There was no significant difference in intraoperative or postoperative recovery parameters between groups.

**Conclusions:** Perioperative administration of dexmedetomidine can exert a protective effect on liver IR injury after hepatectomy. Additional studies are needed to further evaluate postoperative recovery outcomes of dexmedetomidine with different dosing regimens.

## Introduction

Hepatic inflow occlusion by clamping the portal triad (Pringle maneuver), which is traditionally performed to reduce blood loss during hepatectomy, would cause ischemia-reperfusion (IR) injury of the remnant liver after the release of blood flow (Jarnagin et al., 2002; Gurusamy et al., 2009). IR injury is a common but dangerous complication of hepatectomy, especially in patients with underlying liver disease. It can induce local and systemic release of inflammatory mediators and oxygen free radicals, leading to liver or remote organ dysfunction. Numerous strategies have been attempted to reduce IR injury after liver resection, including surgical manipulations, e.g., ischemic preconditioning (Rodríguez et al., 2015) and intermittent hepatic inflow occlusion (Petrowsky et al., 2006), as well as pharmacological intervention, such as anesthetic or sedative agents (Abu-Amara et al., 2009; Li et al., 2016). Although many of them exhibit a protective effect in laboratory and/or clinical researches, agents that commonly used in anesthesia are more preferred as they don’t require additional surgical procedures and won’t prolong the overall time of operation.

Dexmedetomidine, a highly selective α_2_-adrenoceptor agonist, is generally used as an anesthetic adjuvant during surgery and a sedative agent in intensive care unit (ICU). It can offer satisfactory sedation without respiratory depression or hemodynamic instability and exhibit intraoperative anesthetic-sparing effect. It has a theoretical advantage in organ protection, which has been studied in multiple organ systems (Dahmani et al., 2005; Okada et al., 2007; Kundra et al., 2018; Liu et al., 2018; Si et al., 2018; Peng et al., 2020). Animal experiments showed that dexmedetomidine decreased hepatic IR induced oxidative stress and inflammatory responses in liver, serum, and other remote organs (Sahin et al., 2013; Tüfek et al., 2013; Kucuk et al., 2014). Several clinical studies in recent years also proved the hepatoprotective properties of dexmedetomidine against IR injury in patients receiving hepatectomy (Wang et al., 2014; Taman and Elhefnawy, 2019; Zhang et al., 2020). Although most of these researches yield a positive outcome, a comprehensive meta-analysis is still needed to fully evaluate the protective effect of dexmedetomidine on hepatic IR injury.

Therefore, in this study, we conducted a meta-analysis of available randomized controlled trials (RCTs) to systematically discuss the efficacy and safety of perioperative dexmedetomidine use in reducing IR injuries associated with hepatectomy, in order to provide evidence for new strategy of preventive treatment.

## Methods

This meta-analysis was conducted in accordance with the PRISMA (Preferred Reporting Items for Systematic Reviews and Meta-Analyses) statement (Moher et al., 2015). It was prospectively registered on PROSPERO (CRD42020212072). The PRISMA checklist was exhibited in Supplementary File S1.

### Literature Search Strategy

We searched Cochrane Library (Cochrane Center Register of Controlled Trials), MEDLINE, EMBASE, ClinicalTrials.gov, Web of Science, China National Knowledge Infrastructure (CNKI), WanFang, and SinoMed for eligible studies from inception to March 31, 2021. The search keywords were “dexmedetomidine”, “hepatectomy”, “hepatic”, “liver” and “resection”. The search query is given in Supplementary Table S1. Manual searches of references in systematic reviews and included studies were conducted to identify additional qualified researches.

### Inclusion Criteria

We included studies meeting the following criteria: 1) Study design: RCTs; 2) Participants: patients who were scheduled for hepatic surgery irrespective of original liver diseases or Child-Pugh Score; 3) Interventions and Comparisons: comparing dexmedetomidine treatment versus another pharmacological intervention, irrespective of the dose, time, or pharmacological class of the administered drug; 4) Outcomes: perioperative liver function is included as an outcome with available data.

### Exclusion Criteria

RCTs without English abstracts were excluded.

### Outcome Measures

The primary outcome was postoperative liver function as measured by serum concentration of alanine aminotransferase (ALT), aspartate aminotransferase (AST) and total bilirubin (TBIL).

Secondary outcomes included biomarkers of systematic oxidative stress and inflammatory response, postoperative function of remote organs, intraoperative and postoperative recovery parameters. Oxidative stress was indicated by serum malondialdehyde (MDA) level and superoxide dismutase (SOD) activity. Tumor necrosis factor-α (TNF-α) and interleukin-6 (IL-6) were used for evaluation of inflammatory response. Renal function was assessed by urea nitrogen (BUN) and creatinine (Cr); intestinal injury was evaluated by serum diamine oxidase (DAO) activity. Intraoperative parameters included operation time, blood loss and number of patients transfused. Postoperative recovery variables included ventilator support time and length of hospital stay (LOS).

### Data Extraction and Quality Assessment

Two investigators (YH and RW) independently performed the literature retrieval, data extraction and quality assessment. Disagreements were resolved through consensus or by consulting a third author (XL). Extracted data included general study information (e.g., year of publication, country, details regarding interventions and outcomes), baseline data of study participants (e.g., age, gender, original diseases, Child-Pugh score, ASA-status), primary and secondary outcomes. We used the Cochrane risk of bias tool (Higgins et al., 2011) to assess the quality of included studies.

### Statistical Analysis

Continuous data were entered into REVMAN 5.3 (Cochrane Collaboration, Oxford, United Kingdom) for meta-analysis and analyzed using weighted mean difference (WMD) with 95% confidence interval (CI). Data unsuitable for meta-analysis were reported narratively. Heterogeneity across studies was assessed by the Cochrane *χ* ^*2*^ test and *I* ^*2*^ statistic. The random effect model was applied for pooled results with significant statistical heterogeneity (*I* ^*2*^ > 50%); otherwise, the fixed effect model would be used. Sensitivity analysis was performed in Stata (version 12.0; StataCorp LP) to test the stability of this meta-analysis and detect the source of heterogeneity (Chen et al., 2021).

## Results

### Search Results and Study Characteristics

**FIGURE 1:**
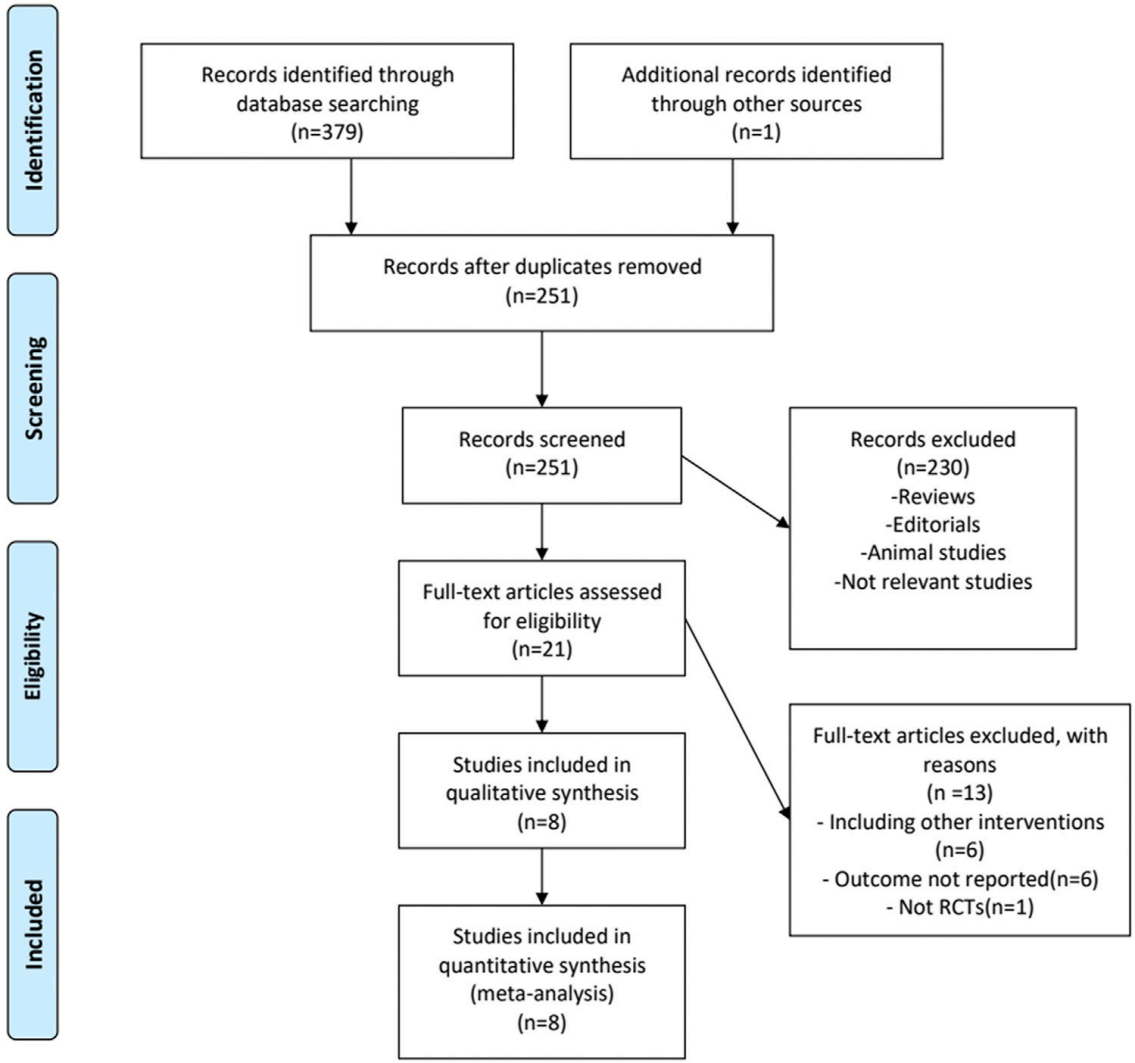
Flowchart of database search and study identification.

A detailed overview of PRISMA flow chart for database searching and study identification is presented in Figure 1. Eight studies enrolling 468 participants were included in this meta-analysis (Wang et al., 2014; Tan et al., 2016; Zhang et al., 2017; Ding et al., 2018; Jiang et al., 2018; Taman and Elhefnawy, 2019; Xing et al., 2020; Zhang et al., 2020). Characteristics of included RCTs and study participants were summarized in Tables 1, 2.

**TABLE 1:** Characteristics of the included RCTs.

Study	Country	Surgery	Intervention and dosage	Treatment duration	Participants	Outcomes
Wang (2014)	China	Hepatectomy	DEX: loading dose 1 μg/kg (10 min), maintain 0.3 μg/kg/h vs. NS	From intubation to the end of surgery	22/22	①②③④⑤⑥⑦
Tan (2016)	China	Hepatectomy	DEX: loading dose 1 μg/kg (15 min), maintain 0.3 μg/kg/h vs. NS	From intubation to the end of surgery	25/25	①②③⑤⑦
Zhang (2017)	China	Hepatolobectomy	DEX: loading dose 1 μg/kg (10 min), maintain 0.5 μg/kg/h vs. NS	10 min before surgery - unclear	53/53	①②③⑦
Ding (2018)	China	Left hemihepatectomy	DEX: loading dose 1 μg/kg (10 min), maintain 0.5 μg/kg/h vs. NS	From induction of anesthesia to the end of surgery	30/30	①②③⑦
Jiang (2018)	China	Precise hepatectomy	DEX: maintain 0.5 μg/kg/h vs. NS	In AICU	20/20	①⑦
Taman (2019)	Egypt	Partial hepatectomy	DEX: maintain 0.3 μg/kg/h vs. NS	From intubation to the end of surgery	25/25	①⑥⑦
Zhang (2020)	China	Hepatectomy	DEX: loading dose 0.5 μg/kg (10 min), maintain 0.5 μg/kg/h vs. NS	Unclear - until liver lobe resection	29/29	①②④⑥⑦
Xing (2020)	China	Partial hepatectomy	DEX: 1 μg/kg (10–15 min, portal vein) vs. DEX: 1 μg/kg (10–15 min, internal jugular vein) vs. NS	10–15 min after isolating the veins	20/20/20	①②③⑦

DEX, dexmedetomidine; AICU, anesthesia intensive care unit; NS, normal saline.

① liver function; ② biomarkers of inflammatory response; ③ biomarkers of oxidative stress; ④ renal function; ⑤ intestinal function; ⑥ postoperative recovery variables; ⑦ intraoperative variables.

**TABLE 2:** Summarized patient characteristic of the included RCTs.

Study	Age (years)	Gender, males	Primary disease	ASA	Child-pugh	Anesthetics	Ischemic time (min)
Wang (2014)	Group C: 30–69; Group D: 27–68	Group C: 71% Group D: 75%	Hepatocellular carcinomas, Intrahepatic cholangiocarcinoma, Intrahepatic bile duct stone	I/II/III	NA	Induction: propofol, fentanyl, cisatracurium; Maintenance: propofol, fentanyl, cisatracurium	Group C: 17.9 ± 5.8; Group D: 19.2 ± 6.4
Tan (2016)	Group C: 47.6 ± 11.4; Group D: 44.6 ± 10.1	Group C: 72% Group D: 76%	Cirrhosis	II/III	NA	Induction: propofol, fentanyl, cisatracurium; Maintenance: propofol, remifentanil, cisatracurium	NA
Zhang (2017)	Group C: 45.38 ± 5.69; Group D: 46.19 ± 5.65	Group C: 56.6% Group D: 54.71%	NA	NA	A/B	Induction: propofol, fentanyl, cisatracurium; Maintenance: propofol, remifentanil, cisatracurium	NA
Ding (2018)	Group C: 54.6 ± 11.4; Group D: 56.6 ± 13.3	Group C: 70% Group D: 76.7%	Primary carcinoma of liver	NA	A/B	Induction: midazolam, sufentanil, rocuronium; Maintenance: propofol, sufentanil, rocuronium, sevoflurane	Group C: 26.9 ± 8.7; Group D: 24.7 ± 10.3
Jiang (2018)	Group C: 44.6 ± 9.2; Group D: 46.7 ± 10.6	Group C: 80% Group D: 70%	NA	II/III	A/B	Induction: midazolam, propofol, fentanyl, vecuronium bromide; Maintenance: propofol, remifentanil, atracurium, sevoflurane	Group C: 51.0 ± 8.1; Group D: 55.1 ± 10.5
Taman (2019)	Group C: 36.4 ± 4.73; Group D: 38.68 ± 4.96	Group C: 76% Group D: 72%	NA	I/II	NA	Induction: propofol, fentanyl, rocuronium; Maintenance: isoflurane, fentanyl, rocuronium	Group C: 20.43 ± 2.58; Group D: 19.61 ± 1.93
Zhang (2020)	Group C: 51.5 ± 11.5; Group D: 52.4 ± 11.8	Group C: 44.8% Group D: 34.5%	Hepatitis, Cirrhosis	I/III	A	Induction: propofol, remifentanil, cisatracurium; Maintenance: propofol, remifentanil, cisatracurium	NA
Xing (2020)	Group C: 44.7 ± 2.7; Group DP: 45.1 ± 3.0; Group DJ: 43.6 ± 3.2	Group C: 55% Group DP: 65% Group DJ: 50%	Intrahepatic bile duct stone, Primary carcinoma of liver	II	A	Induction: midazolam, propofol, sufentanil, rocuronium; Maintenance: propofol, remifentanil, cisatracurium, sevoflurane	Group C: 12.7 ± 1.1; Group DP: 12.3 ± 1.3; Group DJ: 11.9 ± 1.5


Group C, control group (0.9% sodium chloride); Group D, dexmedetomidine group; Group DP, dexmedetomidine group, *via* portal vein; Group DJ, dexmedetomidine group, *via* internal jugular vein; NA, not available.

Three RCTs were published in English (Wang et al., 2014; Taman and Elhefnawy, 2019; Zhang et al., 2020), while the other five were published in Chinese with English abstracts (Tan et al., 2016; Zhang et al., 2017; Ding et al., 2018; Jiang et al., 2018; Xing et al., 2020). These studies all involved one control and one dexmedetomidine intervention group, except one trial which set one control and two intervention groups (same dose of dexmedetomidine given through portal vein and internal jugular vein, respectively) (Xing et al., 2020). In subsequent analysis, this three-arm trial was considered as two independent double-arm trials to facilitate data merging. Dexmedetomidine administration was given intravenously by using a loading dose plus continuous infusion in five studies (Wang et al., 2014; Tan et al., 2016; Zhang et al., 2017; Ding et al., 2018; Zhang et al., 2020), a constant administration rate ranging from 0.3 to 0.5 μg/kg/h in two studies (Jiang et al., 2018; Taman and Elhefnawy, 2019), and a single dose injection in one study (Xing et al., 2020). Dexmedetomidine and control were given before or during the operation except for one study in which they were given during postoperative stay in anesthesia intensive care unit (AICU) (Jiang et al., 2018).

### Study Quality

One study was deemed to be of high risk of bias for inadequate blinding of investigators (single-blind) (Zhang et al., 2020). The other studies were assessed to be of some concerns. The details of quality assessment for included studies are listed in TABLE 3.

**TABLE 3:** Risk of bias evaluation of the included RCTs.

Study	Random sequence generation	Allocation concealment	Blinding in performance	Blinding of outcome assessment	Incomplete outcome data	Selective reporting	Other bias	Total
Wang (2014)	Low	Low	Low	Low	Low	Low	Unclear	SC
Tan (2016)	Unclear	Unclear	Unclear	Low	Low	Low	Unclear	SC
Zhang (2017)	Low	Unclear	Unclear	Low	Low	Low	Unclear	SC
Ding (2018)	Low	Unclear	Unclear	Low	Low	Low	Unclear	SC
Jiang (2018)	Low	Unclear	Unclear	Low	Low	Low	Unclear	SC
Taman (2019)	Low	Low	Low	Low	Low	Low	Unclear	SC
Zhang (2020)	Low	Unclear	High	Low	Low	Low	Unclear	High
Xing (2020)	Low	Unclear	Unclear	Low	Low	Low	Unclear	SC

SC, some concerns.

### Outcome Measures

#### Primary Outcomes

##### Postoperative Liver Function

**FIGURE 2:**
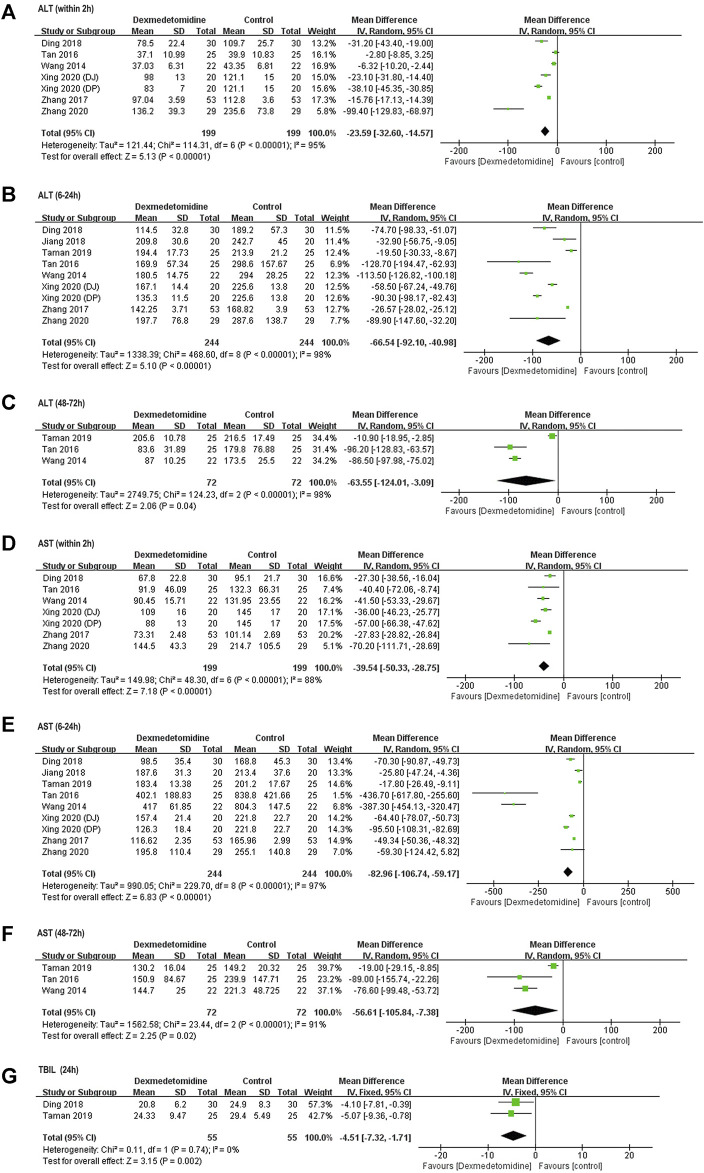
Forest plots for meta-analyses comparing postoperative liver function between dexmedetomidine and control group of participants undergoing hepatectomy. **(A)** ALT (within 2 h after surgery); **(B)** ALT (6–24 h after surgery); **(C)** ALT (48–72 h after surgery); **(D)** AST (with 2 h after surgery); **(E)** AST (6–24 h after surgery); **(F)** AST (48–72 h after surgery); **(G)** TBIL (24 h after surgery).

ALT and AST levels at different time points were reported in all included studies. Compared with baseline (before surgery), postoperative ALT and AST levels were significantly elevated after surgery in control group, indicating the occurrence of hepatic IR injury. ALT and AST levels within 2 h after surgery were markedly decreased in dexmedetomidine group (ALT: WMD = −23.59, 95% CI: −32.60 to −14.57, *p* < 0.00001, *I* ^*2*^ = 95%; AST: WMD = −39.54, 95% CI: −50.33–−28.75, *p* < 0.00001, *I* ^*2*^ = 88%) (Figures 2A,D). Similarly, ALT and AST levels at 6–24 h (ALT: WMD = −66.54, 95% CI: −92.10–−40.98, *p* < 0.00001, *I* ^*2*^ = 98%; AST: WMD = −82.96, 95% CI: −106.74–−59.17, *p* < 0.00001, *I* ^*2*^ = 97%) (Figures 2B,E) and 48–72 h (ALT: WMD = −63.55, 95% CI: −124.01–−3.09, *p* = 0.04, *I* ^*2*^ = 98%; AST: WMD = −56.61, 95% CI: −105.84–−7.38, *p* = 0.02, *I* ^*2*^ = 91%) (Figures 2C,F) after surgery was also reduced by dexmedetomidine. Sensitivity analysis by excluding each included RCT at one time revealed that Taman 2019 was inconsistent with the size of the overall hepaprotective effect of dexmedetomidine (ALT: WMD = −87.57, 95% CI: −98.40–−76.74, *p* = 0.58, *I* ^*2*^ = 0%; AST: WMD = −77.90, 95% CI: −99.55–−56.26, *p* = 0.73, *I* ^*2*^ = 0%) (Supplementary Figure S1), while the other studies were consistent.

TBIL content was reported in two studies (Ding et al., 2018; Taman and Elhefnawy, 2019). Pooled results with a fixed effect model revealed that dexmedetomidine inhibited the increase in TBIL level at 24 h after surgery compared with control (WMD = −4.51, 95% CI: −7.32–−1.71, *p* = 0.002, *I* ^*2*^ = 0%) (Figure 2G).

#### Secondary Outcomes

##### Oxidative Stress

**FIGURE 3:**
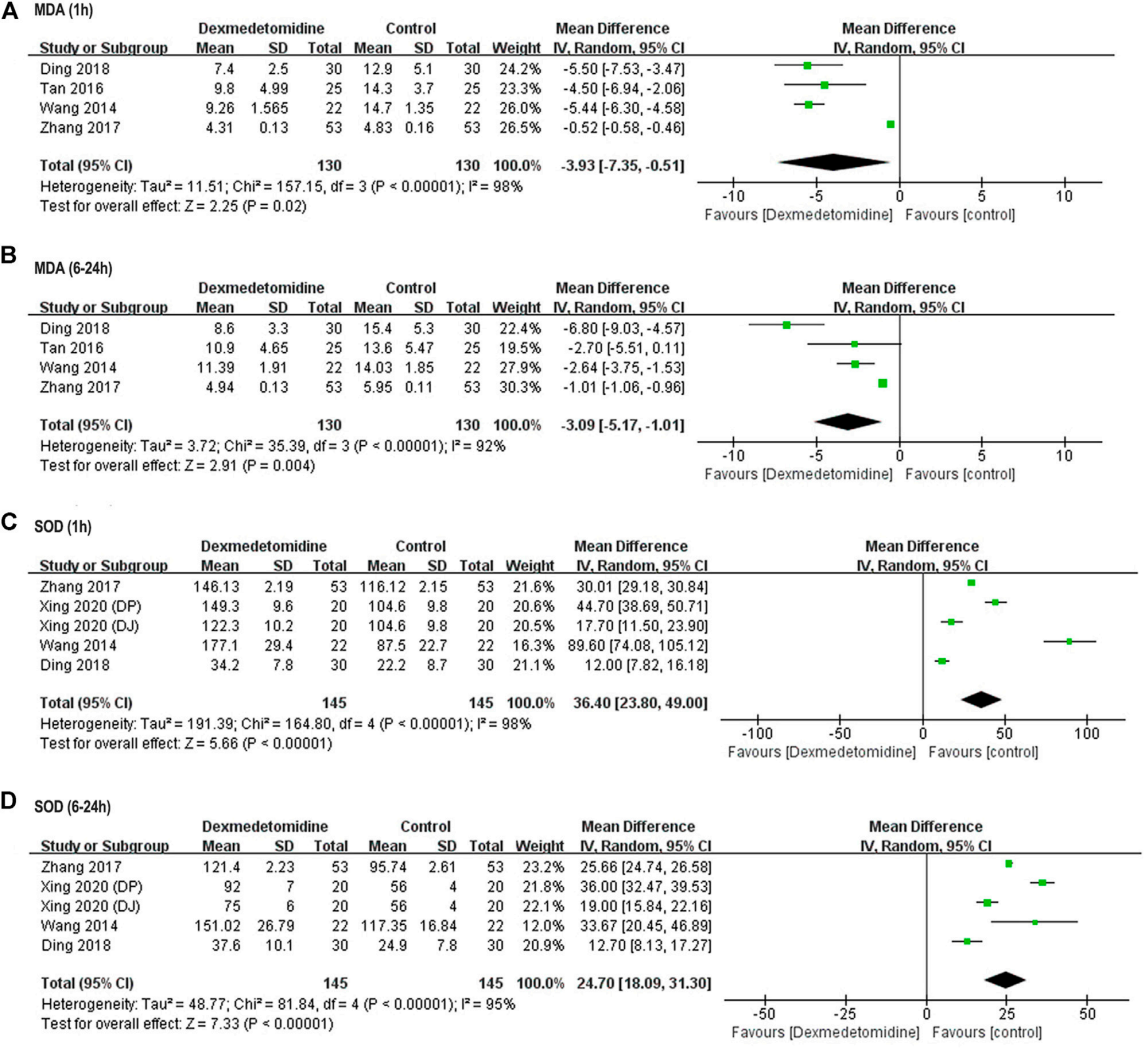
Forest plots for meta-analyses comparing postoperative oxidative stress between dexmedetomidine and control group of participants undergoing hepatectomy. **(A)** MDA (1 h after surgery); **(B)** MDA (6–24 h after surgery); **(C)** SOD (1 h after surgery); **(D)** SOD (6–24 h after surgery).

Perioperative serum MDA and SOD concentration was reported in four studies involving 260 (Wang et al., 2014; Tan et al., 2016; Zhang et al., 2017; Ding et al., 2018) and 270 (Wang et al., 2014; Zhang et al., 2017; Xing et al., 2020) participants, respectively. Significantly elevated MDA level and reduced SOD activity were observed in control group after surgery among all these studies. Postoperative MDA level was markedly reduced (1 h: WMD = −3.93, 95% CI: −7.35–−0.51, *p* = 0.02, *I* ^*2*^ = 98%; 6–24 h: WMD = −3.09, 95% CI: −5.17–−1.01, *p* = 0.004, *I* ^*2*^ = 92%) (Figures 3A,B), while SOD activity was significantly elevated (1 h: WMD = 36.40, 95% CI: 23.80–49.00, *p* < 0.00001, *I* ^*2*^ = 98%; 6–24 h: WMD = 24.70, 95% CI: 18.09–31.30, *p* < 0.00001, *I* ^*2*^ = 95%) in dexmedetomidine group (Figures 3C,D).

##### Inflammatory Response

**FIGURE 4:**
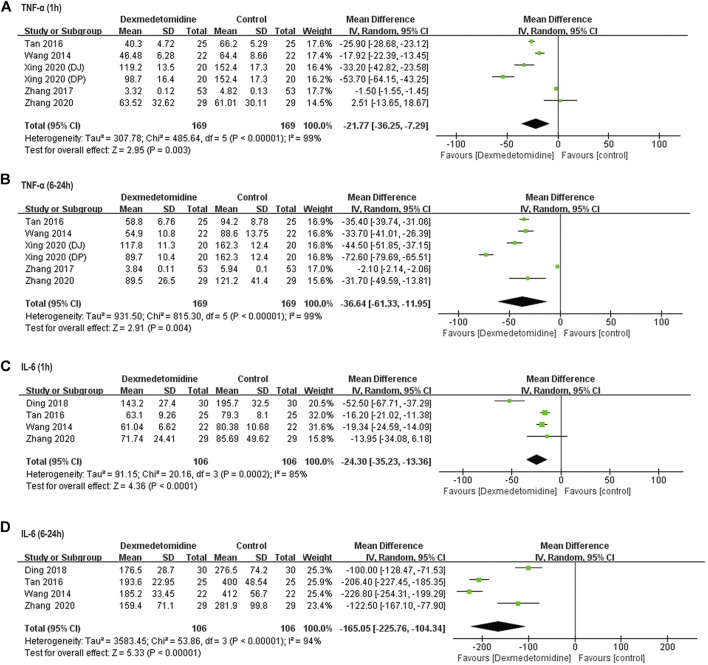
Forest plots for meta-analyses comparing postoperative biomarkers of the inflammatory response between dexmedetomidine and control group of participants undergoing hepatectomy. **(A)** TNF-α (1 h after surgery); **(B)** TNF-α (6–24 h after surgery); **(C)** IL-6 (1 h after surgery); **(D)** IL-6 (6–24 h after surgery).

TNF-α and IL-6 concentration at different time points was reported in five (Wang et al., 2014; Tan et al., 2016; Zhang et al., 2017; Xing et al., 2020; Zhang et al., 2020) and four (Wang et al., 2014; Tan et al., 2016; Ding et al., 2018; Zhang et al., 2020) studies enrolling 318 and 212 participants, respectively. In all these studies, TNF-α and IL-6 level was significantly increased after surgery in control group. However, dexmedetomidine administration decreased TNF-α release from 1 to 6–24 h after surgery (1 h: WMD = −21.77, 95% CI: −36.25–−7.29, *p* = 0.003, *I* ^*2*^ = 99%; 6–24 h: WMD = −36.54, 95% CI: −61.33–−11.95, *p* = 0.004, *I* ^*2*^ = 99%) (Figures 4A,B). Similarly, IL-6 level was also reduced at the same time (1 h: WMD = −24.3, 95% CI: −35.23–−13.36, *p* < 0.0001, *I* ^*2*^ = 85%; 6–24 h: WMD = −165.05, 95% CI: −225.76–−104.34, *p* < 0.00001, *I* ^*2*^ = 94%) (Figures 4C,D).

##### Postoperative Function of Remote Organs

**FIGURE 5:**
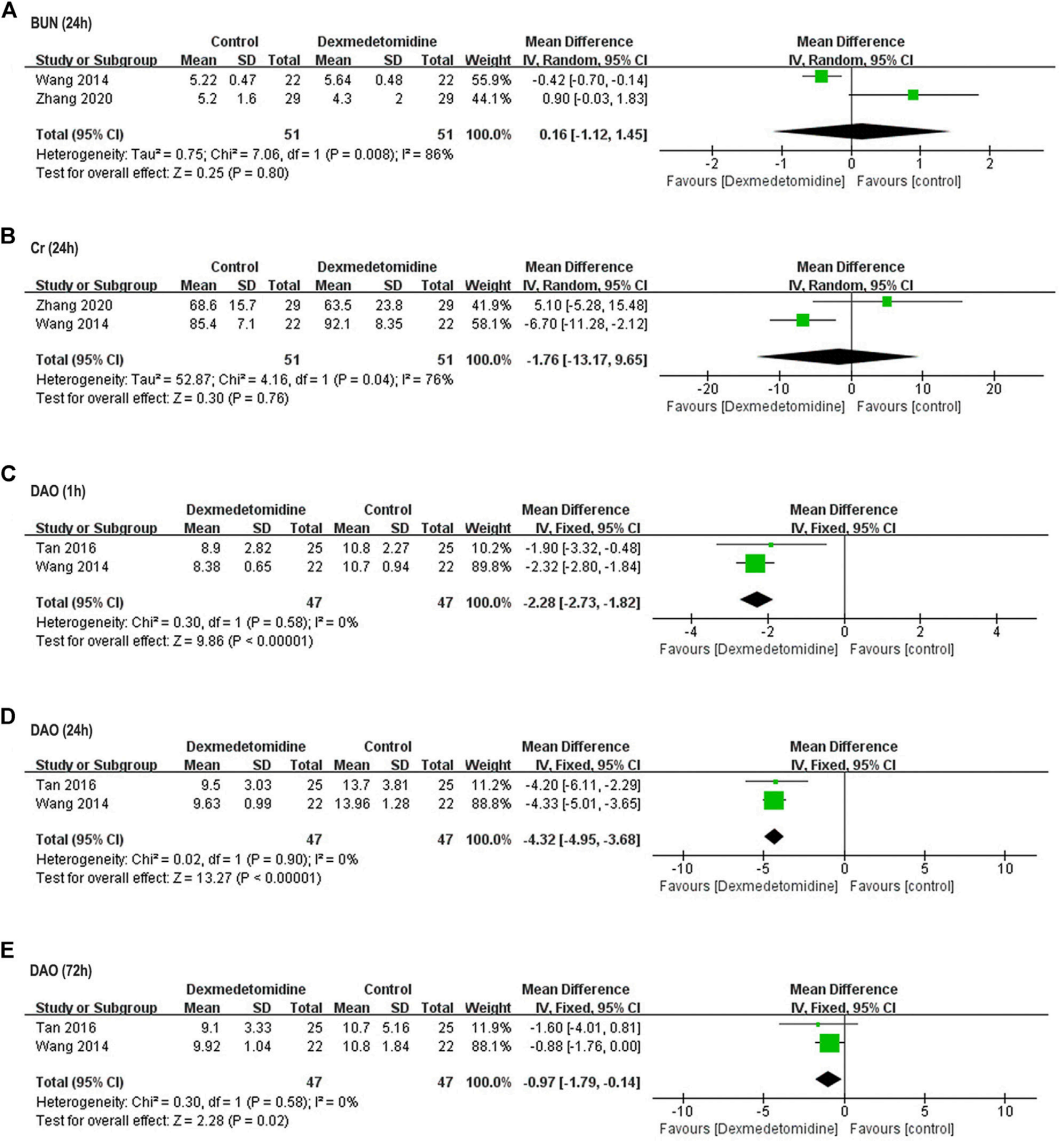
Forest plots for meta-analyses comparing postoperative renal function and intestinal injury. **(A)** BUN (24 h after surgery); **(B)** Cr (24 h after surgery); **(C)** DAO (1 h after surgery); **(D)** DAO (24 h after surgery); **(E)** DAO (72 h after surgery).

Two studies including 102 participants (Wang et al., 2014; Zhang et al., 2020) reported perioperative BUN and Cr concentration. In one study, BUN and Cr level at 24 h after surgery was significantly higher compared with baseline in control group, and dexmedetomidine reversed this elevation; in the other study, postoperative BUN and Cr level remained unaffected in both control and dexmedetomidine group. Taken together, no significant difference was detected in postoperative BUN and Cr level between two groups (BUN: WMD = 0.16, 95% CI: −1.12–1.45, *p* = 0.80, *I* ^*2*^ = 86%; Cr: WMD = −1.76, 95% CI: −13.17–9.65, *p* = 0.76, *I* ^*2*^ = 76%) (Figures 5A,B).

Two studies including 94 participants reported perioperative DAO activity (Wang et al., 2014; Tan et al., 2016). Postoperative DAO activity was significantly elevated in control group of both studies, while dexmedetomidine reversed this elevation within 72 h after surgery (1 h: WMD = −2.28, 95% CI: −2.73–−1.82, *p* < 0.00001, *I* ^*2*^ = 0%; 24 h: WMD = −4.32, 95% CI: −4.95–-3.68, *p* < 0.00001, *I* ^*2*^ = 0%; 72 h: WMD = −0.97, 95% CI: −1.79–−0.14, *p* = 0.02, *I* ^*2*^ = 0%) (Figures 5C–E).

One study reported significant increased CK-MB level in control group compared with that in dexmedetomidine group at 24 h after surgery, but this value was in normal range at baseline and all the postoperative time points in both groups (Wang et al., 2014). Furthermore, there was no declaration of renal failure, myocardial infarction, congestive heart failure, or death after surgery in either group among all studies.

##### Intraoperative Parameters

**FIGURE 6:**
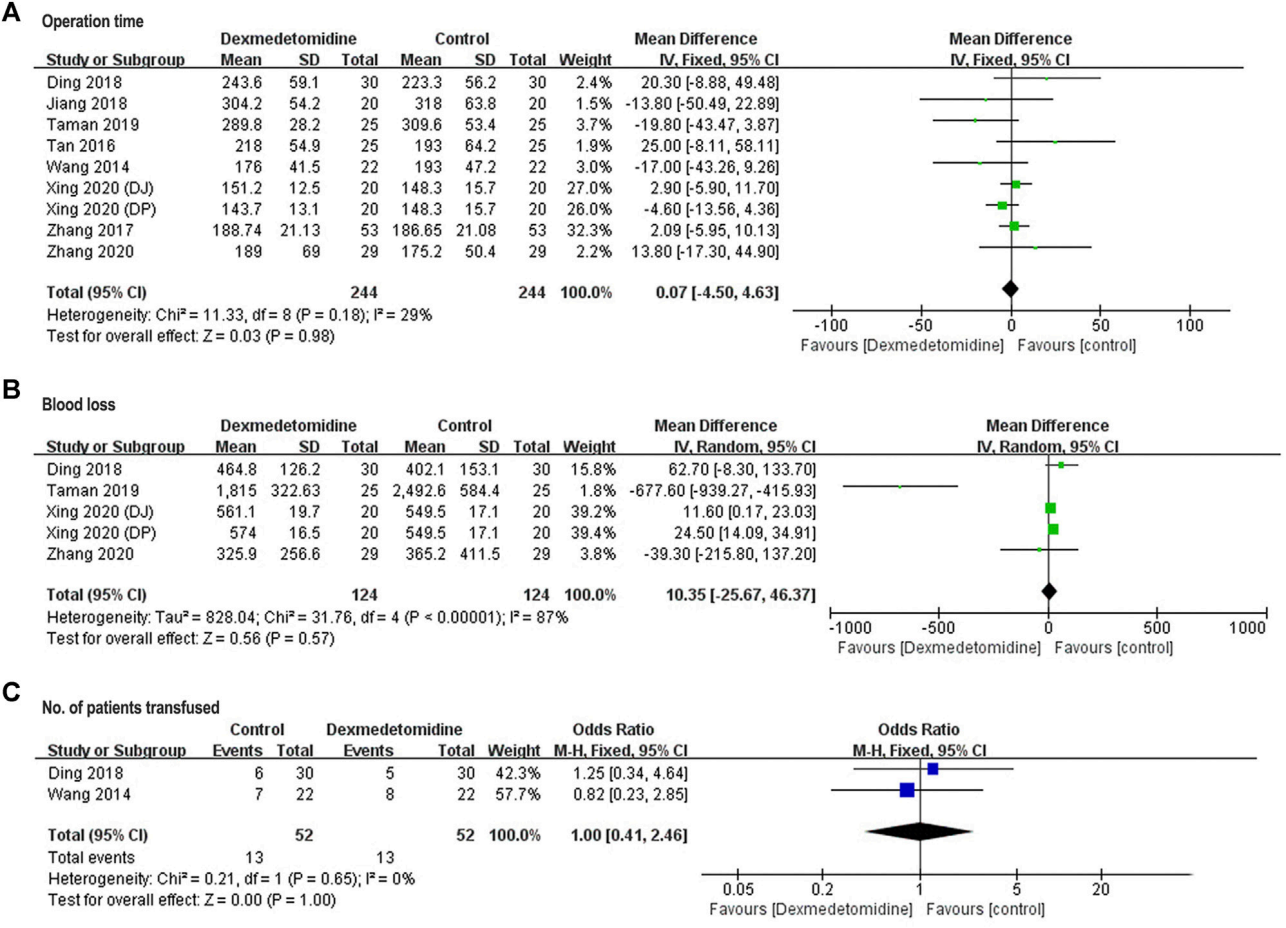
Forest plots for meta-analyses comparing operation time **(A)**, blood loss **(B)**, and No. of patients transfused **(C)**.

Operation time was reported in all the eight studies. Blood loss and number of patients transfused were reported in four studies involving participants (Ding et al., 2018; Taman and Elhefnawy, 2019; Xing et al., 2020; Zhang et al., 2020), and two studies enrolling 104 participants (Wang et al., 2014; Ding et al., 2018). For these three parameters, no significant difference was detected between dexmedetomidine and control group (operation time: WMD = 0.07, 95% CI: −4.50–4.63, *p* = 0.98, *I* ^*2*^ = 29%; blood loss: WMD = 10.35, 95% CI: −25.67–46.37, *p* = 0.57, *I* ^*2*^ = 87%; number of patients transfused: OR = 1, 95% CI: 0.41–2.46, *p* = 1, *I* ^*2*^ = 0%) (Figure 6).

##### Postoperative Recovery Variables

**FIGURE 7:**
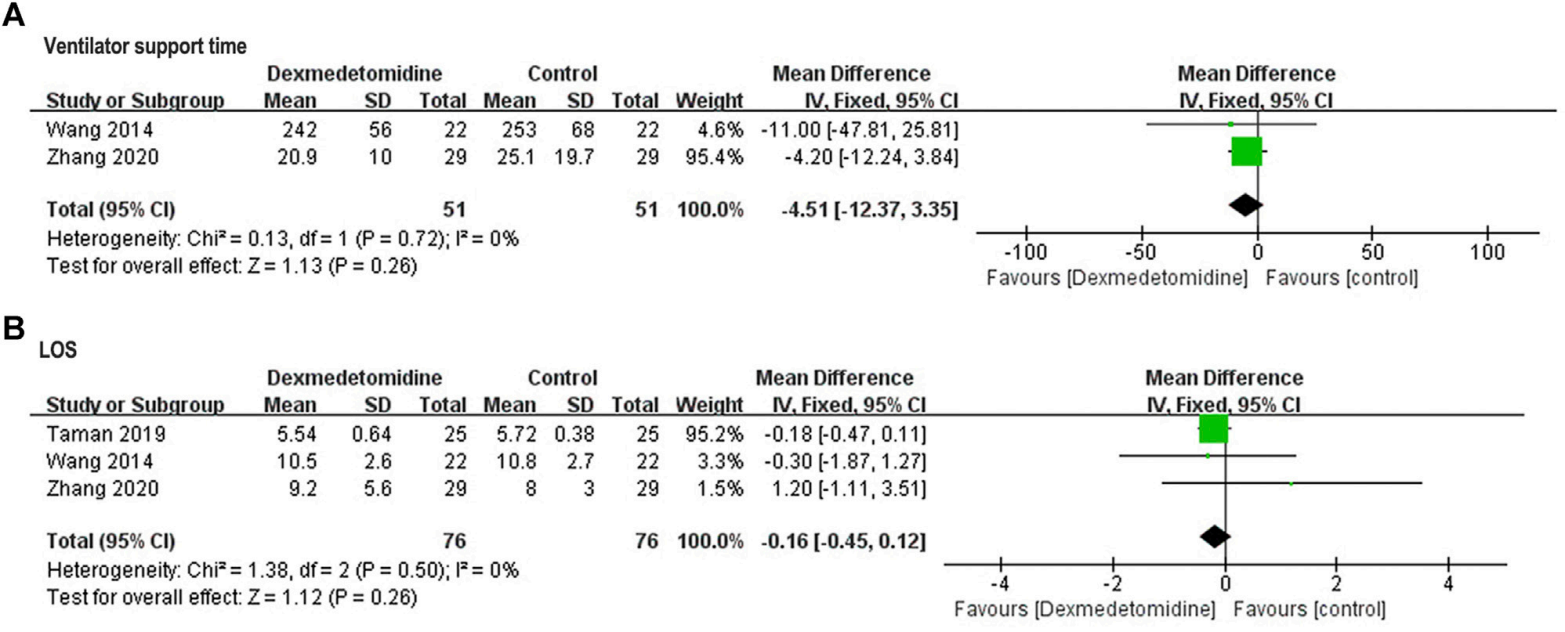
Forest plots for meta-analyses comparing postoperative ventilator support time **(A)** and LOS **(B)**.

Pooled results of postoperative ventilator support time in two studies (Wang et al., 2014; Zhang et al., 2020) (Figure 7A) and LOS in three studies (Wang et al., 2014; Taman and Elhefnawy, 2019; Zhang et al., 2020) (Figure 7B) revealed no significant difference between control and dexmedetomidine group (ventilator support time: WMD = −4.51, 95% CI: −12.37–3.35, *p* = 0.26, *I* ^*2*^ = 0%; LOS: WMD = −0.16, 95% CI: −0.45–0.12, *p* = 0.26, *I* ^*2*^ = 0%).

## Discussion

The present meta-analysis of RCTs demonstrates that perioperative use of dexmedetomidine can produce a protective effect against hepatic IR injury induced by hepatectomy with inflow occlusion. This effect is associated with a systematic decrease in inflammatory response and oxidative stress. Meanwhile, dexmedetomidine also shows a tendency in inhibiting hepatic IR-induced renal and intestinal injury, but has no significant effect on operation time, intraoperative blood loss, and postoperative ventilator support time and LOS. To the best of our knowledge, this is the first meta-analysis that evaluates the efficacy and safety of perioperative dexmedetomidine administration in preventing hepatic IR injury.

Intraoperative blood loss and transfusion is proven to correlate closely with morbidity and mortality after hepatic resection (Jarnagin et al., 2002). Inflow occlusion proceeded to decrease blood loss can induce hepatic IR injury, which may also affect postoperative outcome by activating systemic inflammatory and oxidative stress responses, leading to dysfunction of liver and other remote organs. It has been generally accepted that hepatic IR injury consists of two distinct phases. The initial phase occurs within 2 h postreperfusion and is characterized by Kupffer cell-induced oxidant stress and inflammatory response, resulting in acute hepatocellular injury. The secondary phase appears 6 h or longer after reperfusion and is mainly attributed to neutrophil recruitment induced oxidants and protease release, causing the progression of hepatocyte damage. Therefore, we summarized the observation time points among all studies into three intervals: within 2, 6–24, and 48–72 h after surgery, to evaluate dexmedetomidine effect on IR injury.

In many clinical trials, ALT and AST level have been commonly used as indicators to detect hepatic IR injury and evaluate liver function. In this meta-analysis, all the included studies showed positive hepaprotective effect of dexmedetomidine by reducing postoperative ALT and AST level. TBIL level was also decreased in two studies. Although the elimination half-life of dexmedetomidine is only 2 h, decrease in ALT, AST and TBIL levels can be observed for 24–72 h postoperatively. However, one included study used α-glutathione S-transferase (α-GST) as the primary outcome to detect hepatic IR injury, for its sole distribution in cytosol of centrilobular hepatocytes and rapid response to hepatic IR injury (Zhang et al., 2020). Serum concentration of α-GST was elevated rapidly at 0.5 h postoperatively and returned to normal at 24 h after liver resection, while dexmedetomidine inhibited this elevation at postoperative 0.5 h. Due to relatively high sensitivity and specificity, α-GST may be applied as a parameter to detect hepatic IR injury more rapidly in future clinical trials.

The hepaprotective effect of dexmedetomidine appears to involve attenuation in oxidative stress and inhibition of inflammatory response, which play crucial roles in hepatic IR injury. During the IR process, many chemical substances cause the release of reactive oxygen species (ROS), inducing lipid peroxidation, membrane injury, alterations in ion permeability and enzyme activity, which ultimately leads to cell death. Simultaneously, the inflammatory process begins, significantly increasing the severity of IR injury. In preclinical studies, dexmedetomidine was shown to reduce oxidative activity and increase antioxidant capacity by preventing the elevation of MDA level and increasing paraoxonase activity in the liver and serum of hepatic IR rat model, leading to amelioration of histopathological liver damage (Arslan et al., 2012; Sahin et al., 2013; Tüfek et al., 2013). Meanwhile, dexmedetomidine can suppress the activation of inflammatory pathways, e.g., TLR4-NFκB signaling, and decrease inflammatory mediator level (Wang et al., 2016). Although dexmedetomidine has remarkable binding capacity for all the three subtypes (α_2A_, α_2B_, and α_2C_) of the human α_2_-adrenoceptors, the protective effect against hepatic IR injury may be probably mediated by the activation of α_2A_-adrenoceptor subtype (Wang et al., 2016). Hepatic IR injury induces a systemic response and releases of harmful substances that may affect remote organs. Previous researches showed that the Pringle manoeuvre had a close relationship with intestinal mucosal epithelial injury and represented a predisposing factor for bacterial translocation after liver resection (Erenoglu et al., 2011; Dello et al., 2012). DAO, a degradative enzyme of polyamines highly expressed in the upper villus cells of intestinal mucosa, is generally considered as a specific biomarker of small intestinal mucosal lesions (Luk and Baylin, 1983; Tsujikawa et al., 1999). In our analyses, dexmedetomidine was showed to alleviate postoperative intestinal injury by reducing serum DAO activity. On the other hand, postoperative renal injury appeared only in one study but was not observed in the other. Correspondingly, renoprotective effect of dexmedetomidine was only apparent in one study. Thus, the overall renoprotective effect was not evident according to this meta-analysis. However, previous meta-analysis on the renoprotective effect of dexmedetomidine showed that perioperative use of dexmedetomidine could significantly reduce the incidence of acute kidney injury (AKI) in patients undergoing cardiac surgery (Liu et al., 2018). Thus, additional researches involving renal function as an outcome are needed to further evaluate the protective effect of dexmedetomidine in renal injury induced by hepatic IR. In preclinical studies, the protective effect of dexmedetomidine was also observed in the lungs, kidneys and brain (Tüfek et al., 2013; Yu et al., 2020). Thus, further clinical trials may involve the evaluation of dexmedetomidine effect on more remote organs.

For safety aspects, dexmedetomidine showed no significant alteration on operation time, intraoperative blood loss and number of patients transfused compared to the control group, indicating it is unlikely to cause adverse side effect during operation. Postoperative recovery variables, such as ventilator support time and LOS, were not affect by dexmedetomidine, either. The probable reason may be that dexmedetomidine was administered only during operation or postoperative stay in AICU. The relatively short treatment duration and half-life of dexmedetomidine may prevent its effect from lasting long after surgery.

Among all the included RCTs, dexmedetomidine was administered with different dose regimen and treatment duration. For the limited number of included studies, subgroup analyses were not able to be performed. Previous studies showed that the renoprotective effect of dexmedetomidine differed among dosing groups, but the results are controversial (Balkanay et al., 2015; Liu et al., 2018). Thus, further RCTs with larger sample size and different dexmedetomidine administration regimen will be needed to find the optimal dose of dexmedetomidine treatment.

Our study has several limitations. First, high heterogeneity (*I* ^*2*^ > 50%) existed in most outcomes. It may be attributed to different dose regimen of dexmedetomidine (constant administration rate or loading dose plus maintenance dosage) and sample size of included studies. Due to the limited number of included studies and participants, subgroup analyses were not able to be performed, which made it difficult to detect the source of heterogeneity. Second, included studies were only reported in China and Egypt. Additional studies conducted in other regions with more included participants are needed for systematic evaluation. Third, only two included studies enrolled postoperative parameters as study outcomes, which limited the evaluation of long-term effect of dexmedetomidine.

## Conclusion

In summary, available evidence in the present meta-analysis shows that perioperative use of dexmedetomidine can exhibit a protective effect against hepatic IR injury in patients undergoing hepatectomy. Additional RCTs with larger sample size, different region sites, multiple dosing regimen, and more postoperative outcomes are needed to further evaluate the hepaprotective property of dexmedetomidine against IR injury associated with hepatectomy.

## Data Availability

The original contributions presented in the study are included in the article/Supplementary Material, further inquiries can be directed to the corresponding authors.
